# Body ownership and response to threat

**DOI:** 10.1007/s00426-015-0698-1

**Published:** 2015-08-23

**Authors:** Jing Zhang, Bernhard Hommel

**Affiliations:** 1 Leiden Institute for Brain and Cognition, Leiden University, Leiden, The Netherlands; 2 Center for the Study of Language and Cognition, Zhejiang University, Hangzhou, China; 3 Cognitive Psychology Unit, Institute of Psychology, Leiden University, Wassenaarseweg 52, 2333AK Leiden, The Netherlands

## Abstract

A virtual-reality setup was used to investigate the relationship between perceived body ownership and subjective anxiety, as assessed by an anxiety inventory (SA-I). A pilot study confirmed that synchrony between the participant’s real hand movements and the movements of a virtual effector induced perceived ownership illusions. The illusions were comparable for virtual human hands and virtual cat claws, even though the overall acceptance was greater for human hands. In Experiment 1, participants used the virtual effector to collect coins and avoid knives descending on a screen before anxiety was measured. The level of anxiety increased with synchrony and was higher for human hands than for cat claws, but these two effects were independent. Experiment 2 separated effects of coin catching and knife avoiding by means of a between-participant design. The outcome of Experiment 1 was replicated in the knife-avoiding task but not in the coin-catching task, in which anxiety levels were low and not systematically affected by the type of virtual effector. Taken altogether, our findings suggest that subjective anxiety and ownership are strongly related.

## Introduction

The rubber hand illusion is the experience of an artificial body part as becoming a real body part. This illusion was first reported by Botvinick and Cohen (1998), who placed a rubber hand in front of participants whose corresponding real hand was hidden from sight. When the real hand and the visible rubber hand were stroked in a synchronous fashion, participants reported to experience the rubber hand as being a part of their body. This method is widely used, with various minor and major variations to induce illusions of body ownership (Ide, 2013; Lloyd, 2007; Tsakiris & Haggard, 2005; Zopf, Savage, & Williams, 2010). Among other things, the illusion can also be produced by replacing the rubber hand by a virtual hand that moves synchronously with one’s own hand (Ma & Hommel, 2013; Padilla et al., 2010; Sanchez-Vives, Spanlang, Frisoli, Bergamasco, & Slater, 2010; Slater, Perez-Marcos, Ehrsson, & Sanchez-Vives, 2008).

Perceiving an object as being part of one’s own body has been shown to go along with increased affective reactions to threat directed at this object. Armel & Ramachandran, (2003) repeatedly tapped and stroked participants’ real hidden hand and a rubber hand synchronously (which according to Botvinick and Cohen would induce a sense of ownership for the rubber hand). If the rubber hand was then “injured”, participants displayed a strong skin conductance response (SCR), which is a widely accepted indicator of autonomic arousal (Armel & Ramachandran, 2003; Guterstam & Ehrsson, 2012; Petkova & Ehrsson, 2009). Brain imaging studies also showed that threat to an “owned” rubber hand can induce brain-activity patterns that are commonly associated with anxiety and introspective awareness (in insular and anterior cingulate cortex) and that are also obtained if the participant’s real hand is threatened (Ehrsson, Wiech, Weiskopf, Dolan, & Passingham, 2007).

Yuan & Steed, (2010) measured SCR responses to what they considered threats to a virtual hand. Participants were to play games in a virtual environment by operating a virtual hand or an arrow. During the game, a virtual lamp would fall on the operated virtual effector, which induced a reliable increase in SCR for the virtual hand but not for the virtual arrow. Ma & Hommel, (2013) pointed out that the falling lamp, which only contacted but did not damage the effector, might be taken to represent more of an impact (i.e., a contact-inducing event) than a threat (i.e., a potentially damaging event). To test whether contacting and potentially damaging events trigger different affective states, they combined a standard synchronization technique with the exposure of a virtual hand to either a contact with a ball (which was considered an impact with little damaging potential) or a contact with a cutting knife (which was considered a threat with considerable damaging potential). Their findings show that SCR increased with synchrony (i.e., perceived ownership) in the face of impact but not in the face of threat, which, however, produced elevated SCR levels independently of synchrony/ownership.

The available evidence can thus be taken to suggest that ownership is related to affective reactivity, in the sense that perceived ownership for artificial effectors is associated with stronger affective responses if these effectors are under threat. However, previous studies have used SCR to assess affective reactivity, and employed this measure merely as a convergent measure to assess ownership, while the kind and quality of the affective processes were less relevant. This has several disadvantages. While it is generally accepted that SCR is related to affective reactivity, it is a particularly non-selective, undifferentiated measure that assesses the general level of arousal (Ehrsson et al., 2007; Guterstam, Petkova, & Ehrsson, 2011; Ma & Hommel, 2013) rather than a specific emotion. As a consequence, it is difficult to exclude that SCR effects reflect general motivational attitudes (e.g., preparedness to react) or mere surprise rather than specific emotions. And, even if emotions are involved, it remains unclear which emotions that might be. Several studies have used SCR in the context of conditions that were designed to remind the participant of painful situations (e.g., Armel & Ramachandran, 2003; Yuan & Steed, 2010) and interpreted the thereby induced SCR effects as affective responses. One obvious affective response that such situations are likely to evoke is anxiety, which is why we focused on this emotion in the present study. Indeed, it makes sense to assume that people become particularly anxious if some part of their body is targeted by a threatening event, which suggests that anxiety should be more pronounced for (virtual) effectors that are perceived as part of one’s body. Given that synchrony between one’s own movement and the movement of a virtual effector increases perceived body ownership (Ma & Hommel, 2013; Yuan & Steed, 2010), we thus expected higher anxiety levels under threat to synchronized as compared to unsynchronized virtual effectors.

**Fig. 1:**
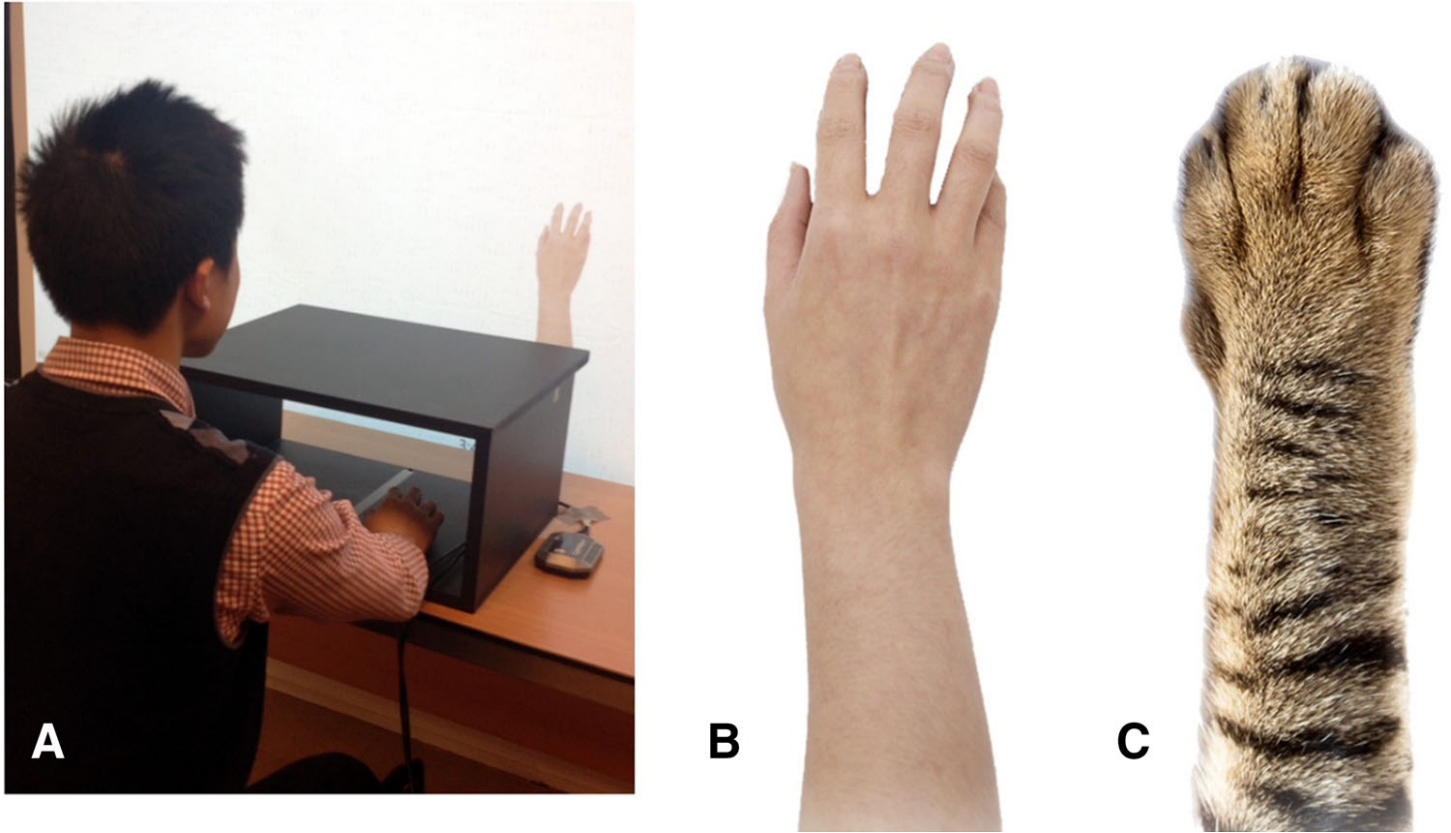
Equipment and virtual images

A second independent variable we considered was the modality of the virtual effector. Similar to the classical rubber hand setup, studies using virtual reality commonly use virtual representations of human hands as candidate body parts. Given that some authors have argued that ownership illusions require a close similarity between the candidate effector and the internal representation of one’s body (e.g., Tsakiris, 2010), this seems to be an obvious choice. However, recent studies have revealed that people can experience body ownership for body-dissimilar effectors as well: Ma & Hommel, (2015a, b) found synchrony-induced increases in ownership perception for virtual balloons and rectangles if participants could control their size, orientation, or color by moving their own hand. They concluded that people may be able to perceive ownership for any event that they can intentionally control. And yet, the findings of Yuan & Steed, (2010) and Ma & Hommel, (2013) suggest that perceived ownership for body-dissimilar effectors may not necessarily translate into the same degree of affective responsivity. With respect to our present study, this suggests that synchrony-induced anxiety under threat may be less pronounced for body-dissimilar than for body-similar effectors. To test that, we manipulated the modality of the virtual effector, which in one condition was a human hand (as in previous virtual-hand experiments) and in another condition was a cat’s claw. The claw was presented in the same orientation as the human hand (see Fig. 1) but clearly different in terms of skin and other details. We were interested to see whether the two effectors would differ in terms of ownership or agency, which we assessed in a pilot study. We were also interested to see whether and how such possible differences would translate into differences in anxiety under threat, tested in the following experiments.

We used an anxiety questionnaire to assess the subjective level of the emotion. The advantage of this method is that it provides direct insight into a specific emotion and the degree to which the participant is experiencing it. However, the disadvantage of this method is that it does not provide a continuous measure, as SCR does, and that the assessment itself takes time and attention. Among other things, this makes it difficult to provide an unbiased assessment of the ownership illusion: filling in an ownership questionnaire first is not unlikely to systematically affect the anxiety level and filling in an anxiety questionnaire first might affect perceived ownership. We, therefore, decided to manipulate perceived ownership by means of the standard synchrony manipulation but to restrict the post-induction assessment to anxiety measurements. As this raises the question whether manipulations were indeed able to induce significant ownership illusions, we first carried out a manipulation check that focused on ownership rather than anxiety. In the following, we first report the outcome of this manipulation check in a pilot study before we turn to the outcomes of two experiments that used the same experimental setup but employed anxiety measures only.

The purpose of Experiment 1 was to assess ownership-related changes in anxiety level. We used synchrony manipulations to induce (if the virtual effector moved synchronously with people’s own hand movements) or not induce (if the movements of the virtual effector were delayed with respect to people’s own movements) perceived ownership (as verified in the pilot study). The general expectation was that people would show higher levels of anxiety if the virtual effector is under threat, especially for conditions that lead to perceived ownership (i.e., with synchrony). We compared the effects for two virtual effectors: one resembling a human hand, as in many previous studies, and another resembling a cat’s claw. As the pilot study showed, less ownership is perceived for a claw than for a human hand (consistent with observations of Guterstam, Gentile, & Ehrsson, 2013; Guterstam et al., 2011; Haans, IJsselsteijn, & de Kort, 2008; Tsakiris, Carpenter, James, & Fotopoulou, 2010), which is why we expected a reduced impact of synchrony on anxiety for claws than for hands.

In Experiment 1, we induced anxiety-relevant threats by having participants engage in a game that required them to use the virtual effector to collect virtual coins and to avoid virtual cutting knives. We hoped that this manipulation would be effective in inducing certain levels of anxiety, especially for “owned” virtual effectors. Experiment 2 replicated these conditions but had each participant play only one of the two games, which allowed us to assess the impact of collecting coins and of avoiding knives on anxiety levels separately.

In Experiments 1 and 2, the levels of anxiety were assessed by means of post-experimental measures only. It is true that additional pre-measures of anxiety would have provided more information and helped reducing the statistical noise resulting from individual differences. However, having participants to report about anxiety at the beginning already would have attracted attention to anxiety being an important dimension for the study. This would have been likely to artificially boost the anxiety level, which in turn would have rendered ceiling effects more likely. Consequently, we assessed anxiety only once per participant, which means that we used post-experimental measures only and that we manipulated all independent variables between participants in all three parts of the study.

## Pilot study

### Method

#### Participants

The participants were 64 undergraduate volunteers (32 female, 32 male) from two universities in Zhejiang, China, who were unfamiliar with the rubber/virtual hand illusion. The age of the participants ranged between 17.92 and 29.96 (M = 20.83, SD = 2.61). All participants were right handed and had normal or corrected-to-normal visual acuity. Ethical approval for this study was obtained from the Zhejiang University ethics committee, and informed written consent was obtained from all participants. Participants were randomly but equally assigned to the four experimental groups.

#### Stimuli and materials

*Experimental setup*. The study was performed in a virtual environment, which was programmed by means of VB.NET. A virtual human hand or cat claw was presented on the screen (see Fig. 1b, c). The mouse was placed in front of the screen but shield by a special box. Participants were asked to observe the movement of the virtual human hand/cat claw while moving the mouse with their right hands (see Fig. 1a). After 3-min moving and observing, participants filled in a 12-item questionnaire which was adopted to evaluate the extent of their virtual effector illusion experience.

*Ownership questionnaire*. We adapted Kalckert & Ehrsson, (2014) 12-statement questionnaire to assess the feelings of agency and ownership to our design (see Table 1). Each statement was scored on a 7 point (−3/3) Likert scale, ranging from −3 for “strongly disagree” to 3 for “strongly agree”. Q1–Q3 are related to the experience of perceiving the hand as one’s “own” hand and Q7–Q9 are directly related to the experience of voluntary control and agency. The remaining questions are sometimes considered control statements, but given their affective quality they may also be suspected to be related to the illusion (e.g., they may well pick up internal conflicts due to an asynchronous temporal relationship between virtual and real hand). We, in any case, report outcomes for these questions as well for the sake of comparability and completeness. For Q10–Q12, we report inverted scales (actual score X-1), as the corresponding questions are phrased in terms of a loss of control and agency. Inverting the scales thus makes the outcomes for the actual agency questions (Q7–Q9) and the agency-related questions (Q10–Q12) semantically more compatible. To work against response strategies, the statements were presented in random order.

**Table 1:** Mean Questionnaire Scores (plus SD) per item

Questionnaire item		Synchronous		Asynchronous	
		Human hand	cat claw	Human hand	Cat claw
1	I felt as if I was looking at my own hand	0.63 (1.996)	−2.25 (1.291)	0.62 (1.668)	−1.88 (1.544)
2	I felt as if the virtual hand was part of my body	0.63 (1.893)	−2.69 (0.704)	0.19 (1.682)	−1.75 (1.390)
3	I felt as if the virtual hand was my hand	1.56 (1.263)	−2.06 (1.482)	−0.19 (1.940)	−1.50 (1.826)
4	It seems as if I had more than on right hand.	−0.88 (1.857)	−2.50 (1.033)	−0.50 (1.414)	−2.19 (1.109)
5	It felt as if I had no longer a right hand, as if my right hand had disappeared	−1.38 (2.094)	−2.31 (1.493)	−1.25 (1.770)	−2.19 (1.601)
6	I felt as if my real hand was turning virtual	−0.25 (1.949)	−2.56 (0.727)	−0.13 (2.125)	−1.19 (2.040)
7	I felt as if I could cause movements of the virtual hand	2.56 (0.727)	2.00 (1.549)	1.19 (1.328)	0.63 (2.187)
8	I felt as if I could control movements of the virtual hand	2.31 (1.078)	2.62 (0.719)	0.94 (1.436)	1.00 (1.789)
9	The virtual hand was obeying my will and I can make it move just like I want it	2.56 (0.727)	2.69 (0.602)	1.00 (1.789)	−0.13 (2.094)
10	I felt as if the virtual hand was controlling my will (inverted)	2.19 (1.109)	2.94 (0.250)	1.81 (1.328)	2.00 (1.592)
11	It seemed as if the virtual hand had a will of its own (inverted)	1.63 (1.455)	2.00 (1.506)	−0.06 (1.482)	0.88 (2.217)
12	I felt as if the virtual hand was controlling me (inverted)	2.19 (1.047)	3.00 (0.000)	1.00 (1.592)	2.62 (0.719)

Items 1–3 are ownership questions, 4–6 are ownership-related questions, 7–9 are agency questions, and 10–12 are agency-related questions

#### Procedure

We used a 2 × 2-factorial between-participants design, which was chosen to avoid possible transfer effects (Zhang, Ma, & Hommel, 2015). The two factors were synchrony (synchronous vs. asynchronous) and modality (human hand vs. cat claw), and each participant was randomly assigned to one of the four conditions. Participants were seated in front of a computer screen, and they could move the mouse with their right hand to control the movement of the virtual effector. After the instruction, participants were exposed to the virtual effector. They could move their own hand (and the mouse) for 3 min, which produced corresponding movements of the virtual effector on the screen with a delay of 0 ms in synchronous conditions or of 350–500 ms (after Shimada, Fukuda, & Hiraki, 2009; Shimada, Qi, & Hiraki, 2010) in asynchronous conditions. Participants were asked to manipulate the mouse by moving their hand. After the completion of this phase, participants filled in the questionnaire.

### Results

**Fig. 2:**
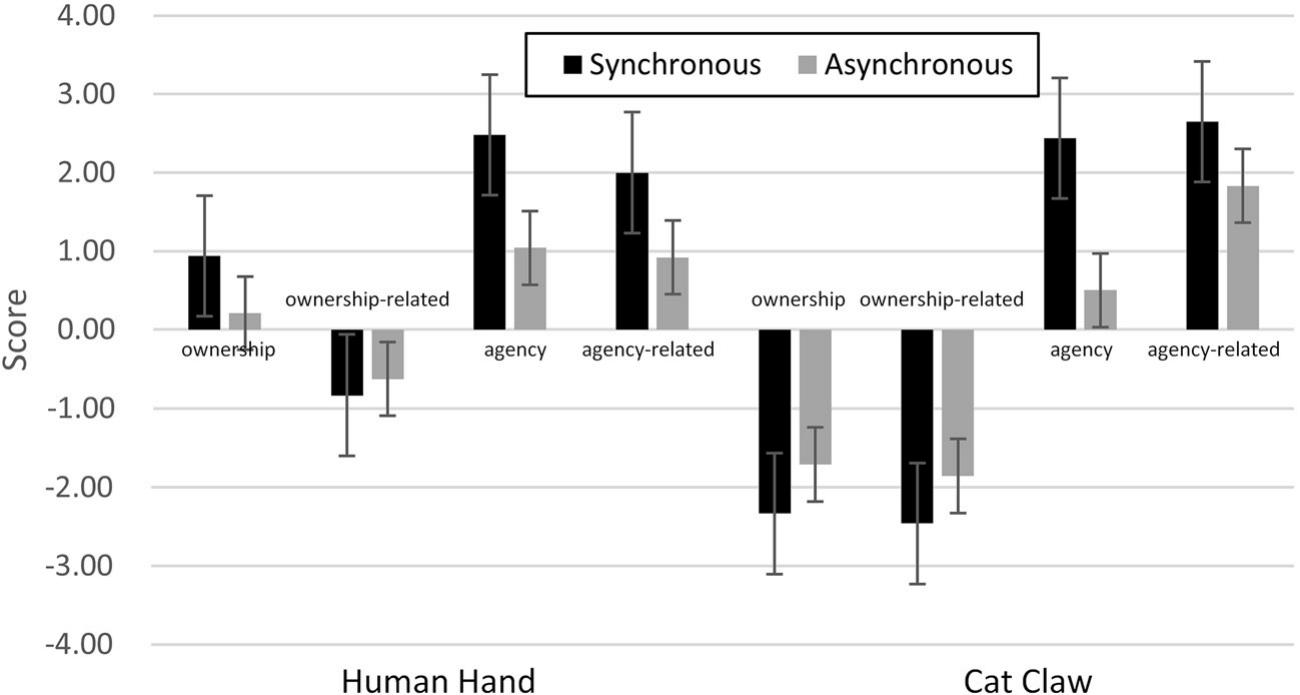
Pilot study: results for ownership, ownership-related, agency, and agency-related judgments

The mean ratings for all four kinds of questions (aggregated for agency, agency-related, ownership, ownership-related) were analyzed with a univariate 2 × 2 ANOVA with the two between-participants factors synchrony (synchronous vs. asynchronous) and effector modality (human hand vs. cat claw) (see Table 1; Fig. 2).

For ownership, there was a significant main effect of modality [*F*(1, 63) = 91.98, *p* < 0.001], while the main effect of synchrony was not significant [*F*(1, 63) = 0.04, *p* = 0.848]. Participants reported a stronger sense of body ownership for the human hand (M = 0.57, SD = 1.21) than for the cat claw (M = −2.02, SD = 1.02), in both synchronous and asynchronous conditions. The interaction between the two factors was significant [*F*(1, 63) = 6.63, *p* = 0.015], indicating that the synchrony effect was more pronounced (and more positive both in terms of the sign of the effect and the general level on the scale) for the human hand than for the cat’s claw. Separate *t* tests revealed that all but the score for the combination of human hand and asynchrony (*p* > 0.5) were significantly different from 0 (*p* < 0.05).

For ownership-related questions, there was also a significant main effect of modality [*F*(1, 63) = 21.21, *p* < 0.001], while neither the main effect of synchrony [*F*(1, 63) = 1.72, *p* = 0.195] nor the interaction between the two factors [*F*(1, 63) = 0.41, *p* = 0.525, respectively] was significant. Participants showed more agreement to ownership-related statements in human hand conditions (M = −0.73, SD = 1.33) than in cat claw conditions (M = −2.16, SD = 1.14), irrespective of synchrony.

For agency, there was a significant main effect of synchrony [*F*(1, 63) = 37.22, *p* < 0.001], while the main effect of modality [*F*(1, 63) = 1.11, *p* = 0.296] and the interaction [*F*(1, 63) = 0.82, *p* = 0.370] were not significant. Participants reported a stronger sense of voluntarily control for synchronously (M = 2.46, SD = 0.71) than for asynchronously moving effectors (M = 0.77, SD = 1.39), irrespective of modality.

For agency-related questions, the main effects of synchrony [*F*(1, 63) = 17.18, *p* < 0.001] and modality [*F*(1, 63) = 11.67, *p* = 0.001] were significant. Participants reported a stronger sense of control over the virtual image (after reversing the scale) in synchronous (M = 2.32 SD = 0.87) than in asynchronous conditions (M = 1.38, SD = 1.09), irrespective of modality. Participants showed lesser loss of control for the cat claw (M = 2.24, SD = 0.98) than for the human hand (M = 1.46, SD = 1.07), irrespective of synchrony. The interaction between the two factors was not significant [*F*(1, 63) = 0.351, *p* = 0.556].

### Discussion

As expected, the synchrony manipulation was successful in inducing the ownership illusion, at least for the human hand. This observation, as well as the fact that the ownership illusion was stronger for the virtual hand than for the virtual claw, confirms that our experimental setup is well suited to manipulate the degree of perceived ownership.

However, while our hand condition replicated previous demonstrations of the virtual-hand ownership illusion, the absence of such an illusion for the cat’s claw can be considered a failure to replicate previous observations of ownership illusion for non-corporeal objects (Ma & Hommel, 2015a). As follow-up studies from our laboratory indicate, this is likely due to the between-participant manipulation of synchrony and modality in the present study. Namely, the strength of ownership illusions is systematically affected by the alternative conditions that given participants are exposed to, suggesting that other conditions provide a kind of mental reference frame for ownership judgments (Zhang et al., 2015). This means that the present between-participant manipulation must be considered relatively conservative as compared to the within-participant manipulations of previous virtual effectors’ studies. Accordingly, we are convinced that a less conservative experimental design would have yielded significant ownership effects for a cat’s claw.

As an aside, it is interesting to see that modality had a strong effect on perceived ownership but not on perceived agency. This suggests that the informational bases for these two judgments do not (entirely) overlap, which is consistent with previous studies showing a discrepancy between (perceived) ownership and (perceived) agency (Kalckert & Ehrsson, 2012, 2014).

## Experiment 1

As explained above, our two actual experiments assessed perceived anxiety. Given that we wanted to avoid influences from the anxiety assessment on perceived ownership and from the ownership assessment on perceived anxiety, we assessed anxiety only. However, with the exception of the second part, we used the exact same experimental setup as in the pilot study, and thus assumed that the manipulations of synchrony and modality would have the same ownership effects than obtained in the pilot study.

### Method

#### Participants

The participants were 96 new undergraduate volunteers (48 female, 48 male) from two universities in Zhejiang, China, who fulfilled the same criteria as in the pilot study. The age of the participants ranged between 17.95 and 29.35 (M = 21.01, SD = 2.53). Ethical approval for this study was obtained from the relevant university ethics committee, and informed written consent was obtained from all subjects. Participants were randomly but equally assigned to the four experimental groups.

#### Stimuli and materials

*Experimental setup*. The setup was the same as in the pilot study, except that there was an additional second part. In this part, participants were exposed to virtual knives and coins falling down the screen, and were to catch the coins and to avoid knives for 2 min by moving their virtual effector accordingly. Every time they caught a coin they were presented with a melodious sound and every time their virtual effector was cut by a knife they were presented with a screaming sound. After finishing this task, participants completed a State-Anxiety Inventory (S-AI; see Appendix) which contains 20 statements related to anxiety. Half of these items (3, 4, 6, 7, 9, 12, 13, 14, 17, and 18) associate higher anxiety with higher scores while the other half associate higher anxiety with lower scores.

#### Procedure

There were two fully crossed experimental between-participants factors: synchrony (synchronous vs. asynchronous) and modality (human hand vs. cat claw), just like in the pilot study. The procedure was very similar to that in the pilot study, the movement between the virtual image and participant’s real hand was either synchronous or asynchronous, and the virtual image was either a human hand or a cat’s claw. The only exception was that, after moving their real hands and watching the movements of the virtual effector on screen for 3 min, participants also performed a catching/avoiding task for 2 additional minutes. In particular, they saw virtual coins and knives falling down from the top of the screen, and they were to catch as many coins, and to avoid as many falling knives as possible. Scores of their performance appeared on the top right corner of the screen during the entire task. Catching a coin or avoiding a knife would add one point while losing a coin or being cut by a knife would lead to the subtraction of one point. At the end of the task, participants were presented with the results of their performances on the screen, and then they filled in the S-AI.

### Results

**Fig. 3:**
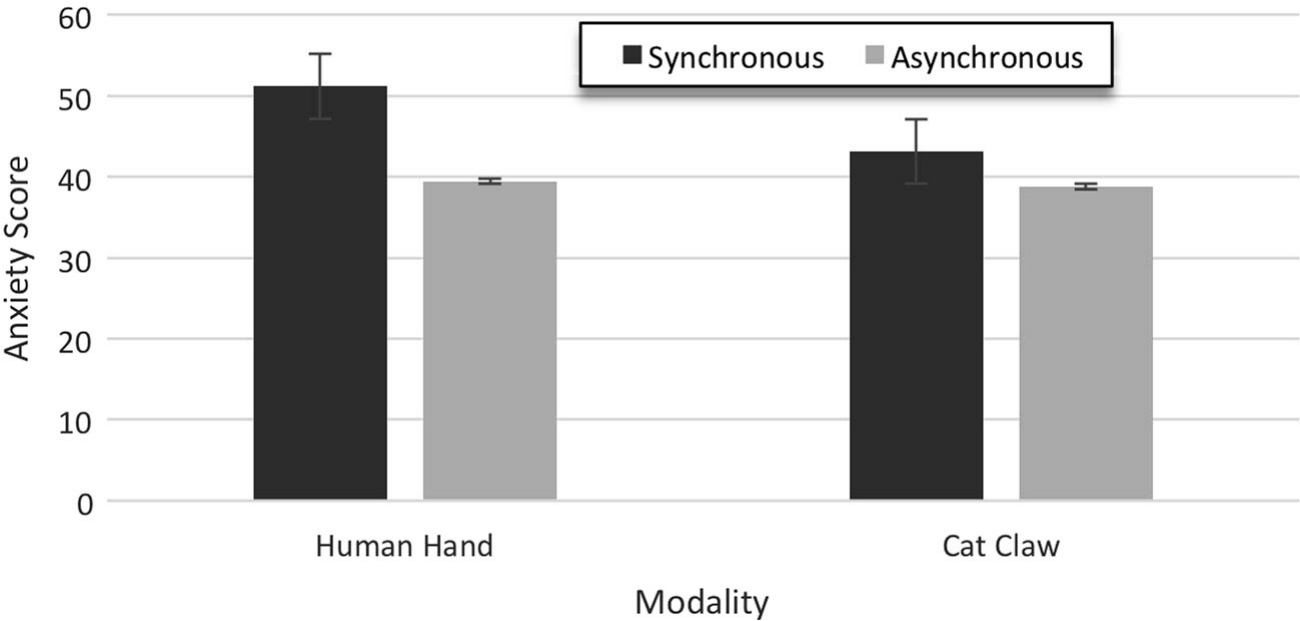
Experiment 1: anxiety results

The anxiety score was calculated by aligning the signs of all scores, so that higher scores indicated more anxiety for all items, and then computing the total. Individual total scores were submitted to a 2 × 2 univariate ANOVA with the factors synchrony (synchronous vs. asynchronous) and modality (human hand vs. cat claw). There were significant main effects of type of synchrony and modality [*F*(1, 95) = 43.69, *p* < 0.001 and *F*(1, 95) = 12.69, *p* = 0.001, respectively], and a significant interaction [*F*(1, 95) = 9.08, *p* = 0.003]. Participants showed more anxiety in synchronous (M = 47.17, SD = 6.43) than asynchronous conditions (M = 39.12, SD = 6.68), and for human hands (M = 45.31, SD = 7.89) than for cat claws (M = 40.98, SD = 6.87), and the synchrony effect was larger for human hands than for cat claws (see Fig. 3).

### Discussion

The aim of Experiment 1 was to test whether the pattern that we obtained in the pilot study for perceived ownership could also be found in explicit anxiety measures. On the one hand, we again obtained a modality effect, which indeed mirrors our ownership findings from the pilot study. That is, people experience more anxiety in the face of threat targeting a virtual effector that they also perceive more ownership for, which also fits with our observation that anxiety is more pronounced with synchrony—the ownership-producing manipulation. On the other hand, however, our experimental design does not allow us to directly relate anxiety to threat. Even though it makes sense to assume that anxiety was more sensitive to the knives than to the coins, we are unable to separate the contributions from these two kinds of events. Experiment 2 was designed to fix that problem by presenting participants with only one kind of these events.

## Experiment 2

The experiment replicated Experiment 1 except that we designed two versions of the game the participants were exposed to in the second part. One version contained descending coins only and participants assigned to this version were to catch them. The other contained descending knives only and participants assigned to this version were to avoid them. This modification of the experimental design created a third between-participants factor (event: catching coins vs. avoiding knives) that was fully crossed with the other two.

### Method

The participants were 96 new undergraduate volunteers (48 female, 48 male) from two universities in Zhejiang, China, who fulfilled the same criteria as in the pilot study. The age of the participants ranged between 17.79 and 27.80 (M = 20.94, SD = 2.34). Ethical approval for this study was obtained from the relevant university ethics committee, and informed written consent was obtained from all subjects. None of those participants were ever engaged in any similar experiment. Participants were randomly but equally assigned to the eight experimental groups.

### Results and discussion

**Fig. 4:**
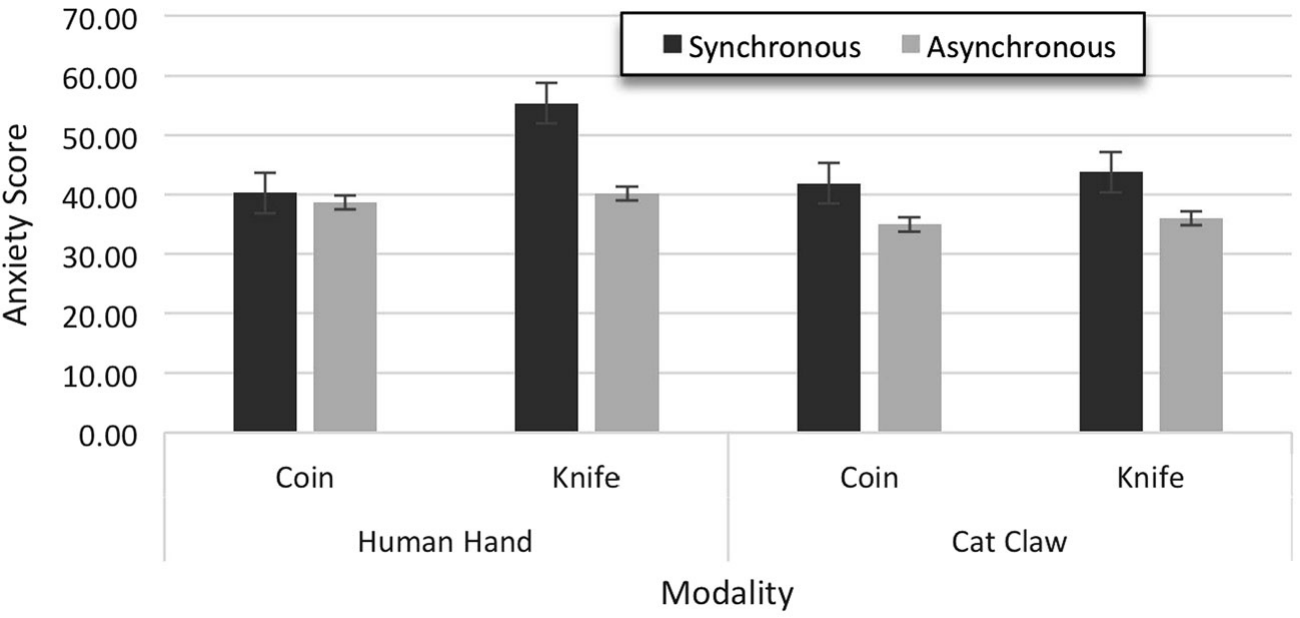
Experiment 2: anxiety results

The mean anxiety scores were submitted to a univariate 2 × 2 × 2 ANOVA with the three between-participant factors synchrony (synchronous vs. asynchronous), modality (human hand vs. cat claw), and event (catching coins vs. avoiding knives) (see Fig. 4). There were significant main effects of synchrony [*F*(1, 95) = 45.59, *p* < 0.001], modality [*F*(1, 95) = 14.83, *p* < 0.001], and event [*F*(1, 95) = 17.71, *p* < 0.001]. Participants showed more anxiety for synchronous (M = 45.29, SD = 7.96) than asynchronous conditions (M = 37.44, SD = 6.15), for human hands (M = 43.60, SD = 8.42) than for cat claws (M = 39.13, SD = 7.17), and when avoiding knives (M = 43.81, SD = 9.91) than when catching coins (M = 38.92, SD = 4.71).

Significant interaction effects between synchrony and event [*F*(1, 95) = 9.60, *p* = 0.003], and between event and modality [*F*(1, 95) = 8.52, *p* = 0.004] were also found, as well as a three-way interaction [*F*(1, 95) = 7.51, *p* = 0.007]. Separate ANOVAs for the two event types showed that the synchrony effect was significant for both the knife condition [*F*(1, 47) = 32.54, *p* < 0.001] and the coin condition [*F*(1, 47) = 13.11, *p* = 0.001], while the modality effect was only significant in the knife condition [*F*(1, 47) = 15.37, *p* < 0.001]. Moreover, synchrony and modality interacted in the coin condition [*F*(1, 47) = 5.16, *p* = 0.028] but not in the knife condition (*p* = 0.072). Additional *t* tests across the two types of events revealed that three of the four conditions were equivalent for both event types (*p* > 0.673) while the combination of synchrony and human hand yielded a much smaller anxiety score in the coin condition than in the knife condition [*t*(22) = 8.93, *p* < 0.001]. That is, the three-way interaction was due to a relative reduction of anxiety for the combination of the virtual hand, synchronicity, and the coin-catching task.

To summarize, the two tasks affected anxiety in different ways: In the knife task, synchrony increased anxiety irrespective of modality, even though the effect was numerically more pronounced for the human hand. In contrast, in the coin task, synchrony had a similar effect for the cat’s claw but not for the human hand, where anxiety levels were comparable for synchronous and asynchronous conditions. This pattern confirms our preliminary conclusion from Experiment 1 that the relation between synchrony (and, by inference: ownership) and anxiety is stronger for the more dangerous knife task.

## General discussion

The main aim of the present study was to investigate the impact of ownership-relevant conditions on perceived anxiety. Taken altogether, our findings suggest three conclusions.

First, people experience more anxiety for threats of effectors that they perceive as part of their own body (cf., Guterstam et al., 2011). While our experimental approach does not speak to the underlying direct causal connections, we systematically find increased anxiety scores in conditions with synchronous relationships between virtual effectors and real hands. As these conditions also increase the perception of ownership, it makes sense to assume that the perception of ownership and the experience of anxiety are based on at least partly overlapping information.

Second, people experience more anxiety for threats of effectors that look more similar to their own hand. The lack of such similarity does not prevent them from experiencing agency, as our pilot study has shown, but it does lead to reduced anxiety even in synchronous conditions. This suggests that pre-existing internal representations of one’s body mediate the experience of anxiety, which fits with claims that self-perception integrates exogenous intersensory information with a more stable internal body image (Synofzik, Vosgerau, & Newen, 2008; Tsakiris, Longo, & Haggard, 2010). However, given that even the cat’s claw produced significant ownership effects, our findings do not support claims that the perception of body ownership is restricted to effectors or objects that resemble one’s own real body parts (Tsakiris et al., 2010). Rather than seeing top-down factors as censoring bottom-up information, our findings can be taken to suggest that bottom-up information (provided through synchrony and related factors) and top-down information (such as general expectations, perceived possibility, and biases) are integrated into a coherent percept.

Third, pronounced, systematic effects on anxiety are restricted to plausible threats. As compared to the knife condition, the coin condition produced rather low anxiety levels overall, which moreover were only mildly affected by synchrony and not sensitive at all to modality. Given that collecting coins is likely to induce some affect and arousal, it is possible that SCR measures would have been more sensitive to pick up (general, arousal-related) affective processes in the coin task. In any case, inducing substantial increases of subjective anxiety seem to require a “true” (even if virtual) threat to an effector that is perceived as part of one’s body. Along these lines, a comparison of the outcomes of Experiment 1 and 2 suggests that the anxiety pattern that we obtained in Experiment 1 was driven by the knife-avoiding part of the task but not by the coin-catching part.

Taken altogether, our findings suggest that the degree of subjective anxiety that people experience when a virtual effector is under threat is perfectly predicted by the degree to which the particular circumstances evoke ownership illusions. Minimally, this suggests that perceptions/judgments of ownership and anxiety are based on overlapping information. Exciting situations that, however, do not threaten the “physical integrity” of the virtual effector have a mild and rather nonspecific impact on anxiety, which confirms that the major part of the anxiety effect is threat specific. This observation is consistent with previous findings that mere impacts and actual threats affect SCR levels in different ways (Ma & Hommel, 2013). It is also consistent with previous demonstrations of a positive relationship between perceived ownership and SCR responses under threat (Armel & Ramachandran, 2003; Yuan & Steed, 2010) but goes beyond these demonstrations by showing a specific effect on anxiety. This does not rule out effects on other factors that are known to impact SCR, such as surprise or motivation—the demonstration of which would require the employment of more specific measures than SCR. Separating these effects seems useful and important on the way to a better understanding of the functional and neural mechanisms underlying the perception of body ownership.

## Appendix

See Table 2.

**Table 2:** Questions from state-anxiety inventory

1	I feel calm	11	I feel self-confident
2	I feel safe, secure	12	I feel nervous, irritable
3	I feel tense, nervous	13	I feel scared, alarmed, afraid
4	I feel stressed	14	I feel uncertain
5	I feel peaceful, good about myself	15	I am relaxed, at ease
6	I feel upset, overwhelmed	16	I am satisfied
7	I worry over possible misfortunes	17	I am anxious, worried
8	I feel happy	18	I feel disconcerted, disoriented
9	I feel frightened	19	I feel collected, composed
10	I feel at ease	20	I feel pleasant, in a good mood
